# Optical Identification of Fruitfly Species Based on Their Wingbeats Using Convolutional Neural Networks

**DOI:** 10.3389/fpls.2022.812506

**Published:** 2022-06-03

**Authors:** Ioannis Kalfas, Bart De Ketelaere, Tim Beliën, Wouter Saeys

**Affiliations:** ^1^Department of Biosystems, Faculty of Bioscience Engineering, MeBioS, KU Leuven, Leuven, Belgium; ^2^Zoology Department, pcfruit vzw, Sint-Truiden, Belgium

**Keywords:** insect recognition, convolutional neural network, pest management, automatic monitoring system, wingbeat analysis, wingbeat frequencies, optical sensing and sensor, deep learning

## Abstract

The spotted wing Drosophila (SWD), *Drosophila suzukii*, is a significant invasive pest of berries and soft-skinned fruits that causes major economic losses in fruit production worldwide. Automatic identification and monitoring strategies would allow to detect the emergence of this pest in an early stage and minimize its impact. The small size of *Drosophila suzukii* and similar flying insects makes it difficult to identify them using camera systems. Therefore, an optical sensor recording wingbeats was investigated in this study. We trained convolutional neural network (CNN) classifiers to distinguish *D. suzukii* insects from one of their closest relatives, *Drosophila Melanogaster*, based on their wingbeat patterns recorded by the optical sensor. Apart from the original wingbeat time signals, we modeled their frequency (power spectral density) and time-frequency (spectrogram) representations. A strict validation procedure was followed to estimate the models’ performance in field-conditions. First, we validated each model on wingbeat data that was collected under the same conditions using different insect populations to train and test them. Next, we evaluated their robustness on a second independent dataset which was acquired under more variable environmental conditions. The best performing model, named “InceptionFly,” was trained on wingbeat time signals. It was able to discriminate between our two target insects with a balanced accuracy of 92.1% on the test set and 91.7% on the second independent dataset. This paves the way towards early, automated detection of *D. suzukii* infestation in fruit orchards.

## Introduction

*Drosophila suzukii* (Matsumura), the spotted wing *Drosophila* (SWD), is a major invasive fruit pest which is native to Western Asia, but has spread to many countries around the world. It was first spotted in Southern Europe in 2008 (Rasquera, Spain) and in the following years it spread to the majority of European countries across a wide range of environmental conditions and climates (Mortelmans et al., 2012; Asplen et al., 2015). Unlike the majority of other Drosophilidae, *D. suzukii* lays its eggs in healthy ripening fruits rather than damaged or overripe ones, thus creating special problems to growers. The host range of SWD includes mainly soft-skinned fruits and it is quite broad, having now been documented in cherries, peaches, nectarines, plums, persimmons, strawberries, grapes, blackberries, blueberries, raspberries, pluots, figs, and several other fruit crops, as well as a wide variety of non-crop host plants (Walsh et al., 2011; Kenis et al., 2016; Tait et al., 2021). Damage in fruit production by SWDs ranges from negligible to 80% crop loss (Dreves et al., 2009; Lee et al., 2011; Walsh et al., 2011; Asplen et al., 2015; Potamitis and Rigakis, 2015; Klick et al., 2016; Farnsworth et al., 2017; Yeh et al., 2020). A study looking into revenue losses due to SWD infestation and fruit rejections found that gross revenues decreased by 37% for raspberries and 20% for strawberries in California, United States (Goodhue et al., 2011). The spread of *D. suzukii* is quite fast since it is introduced or re-introduced to habitats worldwide *via* global fruit trade and it then moves quickly from one region to another by flying (Rota-Stabelli et al., 2013). Consequently, knowledge of SWD (or similar) population sizes at any given time would be very useful to growers of host crops and parties directly or indirectly affected by the subsequent economic losses since it would provide the ability to assess new possible infestations or the severity of existing ones.

Most traditional monitoring methods require a frequent human intervention to either sample larvae in fruits or identify and count trapped insects. These labor-intensive procedures are time consuming and can be inefficient when dealing with rapid pest invasions. In the case of SWD, their population can double in size in only 4 days (Emiljanowicz et al., 2014) and a single female can produce approximately 3,000 adult descendants within a couple of months (Tochen et al., 2014). Moreover, SWD flies are known to utilize a variety of non-crop hosts and alternative habitats (Dalton et al., 2011; Burrack et al., 2013; Atallah et al., 2014), which makes manual monitoring methods progressively more challenging and inefficient as the number of necessary inspection areas and field types increase. Besides, the high activity season of the SWD varies and lasts quite long, ranging from early July until late December according to studies conducted in the eastern part of the United States (Pelton et al., 2016; Guédot et al., 2018) as well as Europe (Clymans et al., 2019; Tait et al., 2021). Hence, a necessity for more automated monitoring systems of pest insect populations arises.

Automatic monitoring systems of pests can generate timely warnings in real-time and prompt farmers to act if needed. This could also help control the use of insecticides, which create severe negative effects on public health and the environment (Wilson and Tisdell, 2001; European Commission, 2019). By relying on data-derived metrics of pest population sizes, insecticide use could be applied only under certain infestation conditions and not as a precautionary measure. In the past years, several automatic insect traps have been developed (Jiang et al., 2008, 2013; Shieh et al., 2011; López et al., 2012; Lampson et al., 2013; Potamitis et al., 2015; Lima et al., 2020a). The two main approaches that prevail in designing insect monitoring devices are: (1) imaging of trapped insects; and (2) recording a sensor reading of the insect upon entry.

In the first approach, the insects are commonly trapped on a sticky surface which is imaged by a camera. Then, the trapped insects on that surface are counted and identified by using simple computer vision and artificial intelligence (AI) algorithms (Espinoza et al., 2016; Nieuwenhuizen et al., 2018; Lima et al., 2020a). Image-based traps are frequently combined with Convolutional Neural Network (CNN) classifiers and object detectors (Li et al., 2021). For example, Roosjen et al. (2020) used images taken from an unmanned aerial vehicle (UAV) and fed them to CNNs to detect SWD individuals trapped on sticky plates. They demonstrated a rather low area under the precision-recall curve (AUC) of 0.086 for female SWDs and 0.284 for male. When using static images instead, they detected female SWDs with a promising AUC of 0.506 and male SWDs with AUC of 0.603. Thus, despite the success of CNN models in classifying images or detecting objects, systems that employ CNNs still struggle to address challenges that arise in the field, such as varying illumination, blurry images due to insect movement, orientation or crowding, and uncalibrated systems (out of focus cameras, poor color calibration, white balancing, etc.). To overcome some of these challenges, practitioners often apply data augmentation by creating replicas of their original data with visual differences that simulate various real conditions. This way, CNN models learn features that distinguish their target insects from others in multiple different settings. Still, classifying small insects in images remains a challenge even for such complex models, especially for insects that do not have prominent or unique features.

In sensor-based insect traps, often an infrared or optical sensor is placed inside a lure trap to count the number of times a target insect enters, or to capture its wingbeat pattern or produced vibrations to classify it (van Roy et al., 2014; Potamitis and Rigakis, 2015; Potamitis et al., 2017; Lima et al., 2020b; Kalfas et al., 2021; Rigakis et al., 2021). Sensor-based traps are paired with lures, and they can either record events that likely belong to a target insect or capture more complex patterns on which prediction models are built. In two example cases, researchers built a detection system for Red Palm Weevil infestations in trees using bioacoustics signals produced by this insect (Ilyas et al., 2009; Hussein et al., 2010). Bioacoustic signals like calling or courtship sound signals are also recorded using microphones or similar audio recorders to classify insect species (Mankin, 1994; Chesmore, 2001; Raman et al., 2007; Zamanian and Pourghassem, 2017), but these devices are sensitive to wind noise or ambient sounds when deployed in the field. In two different studies, Potamitis et al. (2014, 2015) embedded an optoelectronic sensor in a McPhail-type trap and were able to count and classify fruitfly species by measuring the insects’ wingbeat. Optoelectronic sensors provide several benefits for recording insect biometric data compared to microphones and cameras since they are not influenced by the environmental conditions or the target’s distance from the sensor while recording data (Potamitis et al., 2018). Wingbeat data captured from optical sensors have already been used successfully to classify insect species and with the recent advances in the field of Machine Learning (ML) it has become possible to build high-performing classification systems (van Roy et al., 2014; Chen et al., 2014; Potamitis and Rigakis, 2015; Fanioudakis and Potamitis, 2018). However, strict validation procedures are crucial to avoid that over-optimistic results are obtained with these powerful machine learning techniques. In a previous study involving a rigorous validation strategy, we have shown that CNNs are able to classify wingbeat data of mosquitoes on the genus level, but were less successful at the species level (Kalfas et al., 2021).

Both the *D. melanogaster* (DM) and the *D. suzukii* (SWD) occur in similar habitats with presence of soft-skinned fruits and overlapping high activity seasons. However, unlike SWD, DM poses no considerable threat to fruit crops since it will mainly attack overripe fruit that are already unfit for sale. Hence, a system that can accurately discriminate between the two *Drosophila* genera will be very valuable to estimate the need for crop protection at any given time. Both insect types are very small in size and range between 2 and 4.5 mm in body length, and 2 and 3.5 mm in wing length (Walsh et al., 2011). On average, DMs are slightly smaller than SWDs, but there is substantial overlap between both populations. Using optical sensor recordings of the wingbeats, we aim to overcome the limitations that an in-field camera system would have, dealing with such small insects with similar appearance. As no reports were found on the discrimination of these highly similar inspect species from the Dropsophila genus based on their wingbeat signals, the aim of this study was to train and strictly validate CNN classifiers to discriminate wingbeat signals of the SWD pest from the DM as a stepstone towards automatic in-field pest monitoring.

## Materials and Methods

### Insect Stock Culture

The *D. suzukii* culture used in the laboratory experiments originated from multiple collections of adults in a private garden (Gentbrugge, Belgium, 51°1.522′N, 3°46.093′E). The *D. melanogaster* culture was received from the “Expertise Unit on Educational Provision” (Faculty of Bioscience Engineering, KU Leuven, Belgium). The laboratory colonies were maintained in polystyrene Drosophila vials (Greiner Bio-One^™^ Insect Breeding Conical Container, 217,101) on a cornmeal-yeast-agar diet (42 g/l fresh yeast, *Saccharomyces cerevisiae*, Algist Bruggeman; 55 g/l white table sugar, Suikerraffinaderij Tienen; 90 g/l crushed cornmeal, Aveve; 2 g/l Ethyl 4-hydroxybenzoate 99%, Alfa Aesar; 9 g/l agar powder, VWR chemicals and 910 g/l tap water). The vials were stoppered using foam stoppers (Greiner Bio-One^™^ Ceaprenstop, diameter 36 mm, 330,070) and kept in a plant-growth chamber at 22 ± 1°C, 60 ± 11% RH, and a 16:8 l:D photoperiod.

### Sensor Design

**Figure 1:**
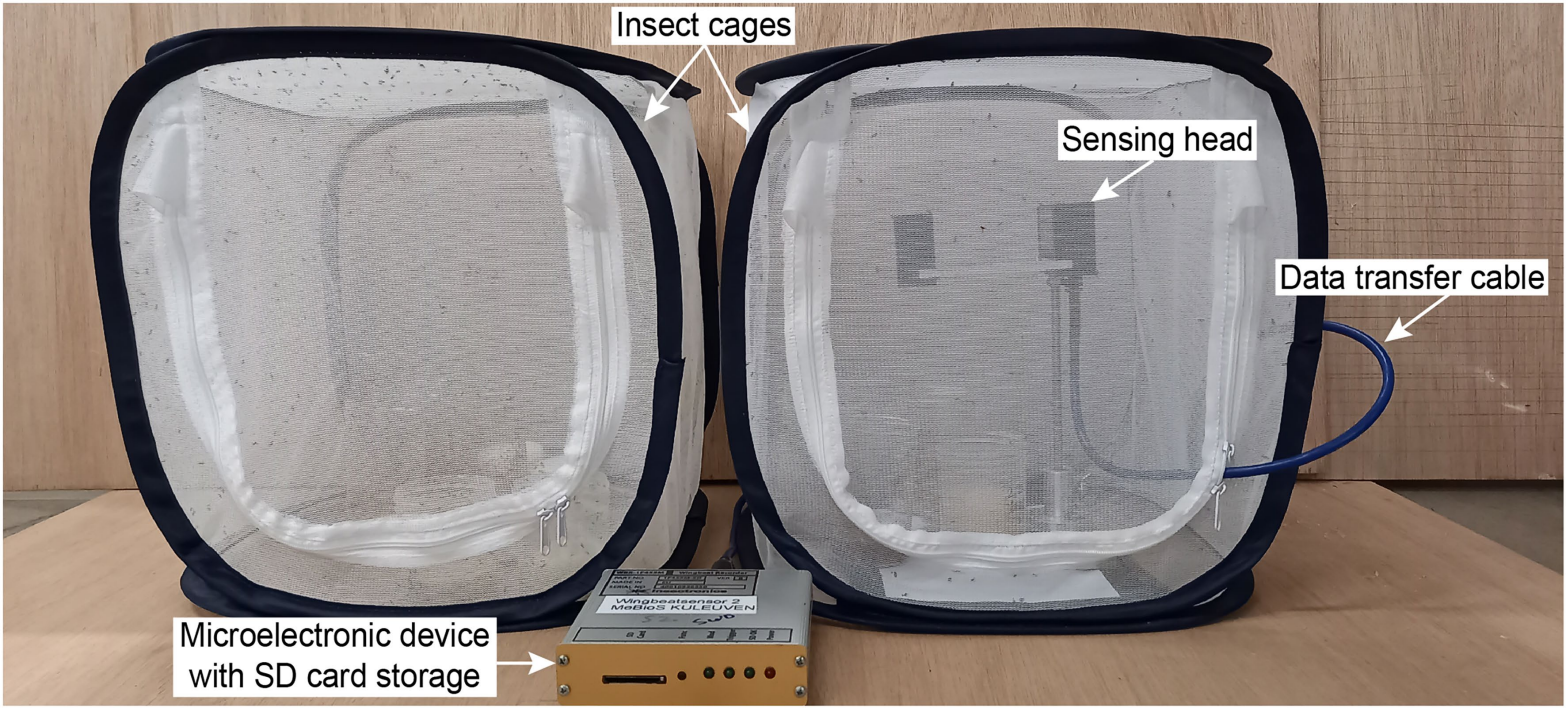
Photograph of the laboratory setup with two insect cages and the wingbeat sensor. The wingbeat sensor consists of a sensing head, a data transfer cable and a microelectronic device with an SD card storage.

The wingbeat sensor consists of two main parts: (a) a sensing head and (b) a microelectronic device that handles how the signals are stored (Figure 1). The sensing head consists of two boards placed opposite to each other, which act as a light emitter and receiver, respectively. As an insect flies between the two boards, it occludes the emitted light with its body and wings. The light receiving board then records a pattern of varying light intensity values which constitutes the wingbeat signal in the time domain. The microelectronic device measures the Root Mean Square (RMS) value of the live signal and contains software that defines the sampling frequency, triggering and storing of wingbeat events (in an embedded SD card). For more details regarding the wingbeat sensor device we refer to Potamitis and Rigakis, 2015 and Kalfas et al., 2021.

### Experimental Setup and Data Collection

**Figure 2:**
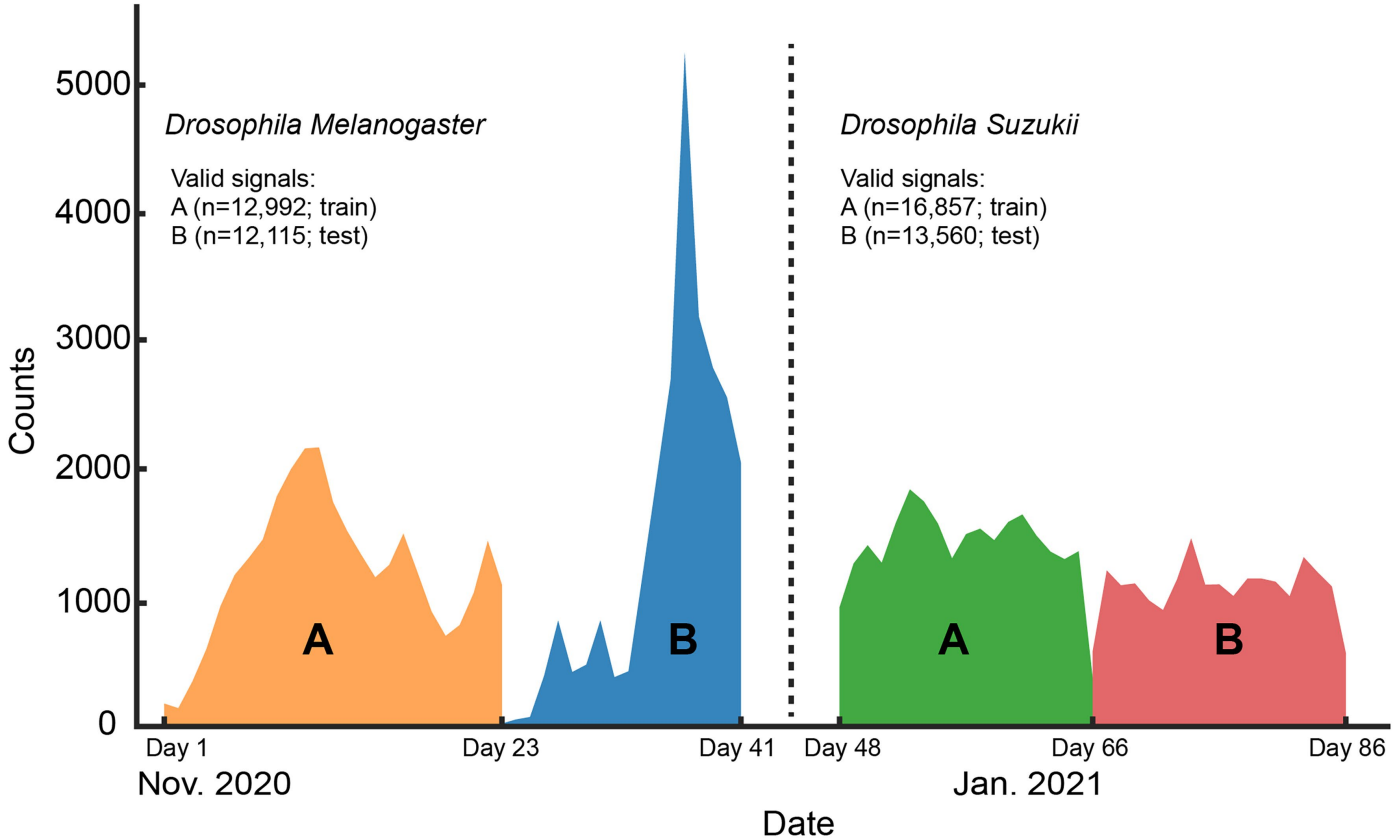
Histogram of the signal counts collected on each day for the whole length of the controlled environment experiment. The number of valid signals per Drosophila species and the data split (train or test) they belong to are shown in the legend.

All wingbeat data were recorded in a laboratory or a climate room by placing an optoelectronic sensor inside spacious insectary cages where either *D. melanogaster* or *D. suzukii* insects were free to fly in (Figure 1). The same sensor device was placed in each insect cage sequentially for a period of 2–3 weeks (Figure 2) until sufficient data were collected for each population, considering that the number of valid signals would be fewer than the total number of signals per population after our data cleaning process. We reared two separate populations per *Drosophila* species (four insect populations in total) and tried to limit the number of insects in each population to around 200–300 individuals. We did not select insects based on their age or sex and new insects kept on hatching from larvae in the food media during the entire experiment. The vials with the food media were replaced once the food was depleted and no new eggs seemed to appear inside.

To collect a dataset of wingbeat signals under controlled conditions (Controlled dataset in Table 1), all insect cages were placed in a “climate room” to have stable environmental conditions. The average temperature in this room was 22 ± 0.6°C and the average relative humidity was 64 ± 5%. During this controlled experiment 99,154 wingbeat signals were recorded across all populations. False triggers and weak signals (with a noisy Power Spectral Density) were filtered out by employing a data cleaning procedure which is explained in “Selected Data Types and Data Cleaning.” The numbers of retained signals are summarized in Table 1.

**Table 1:** The number of signals for the two datasets used in this study (Controlled and Remote-Uncontrolled) and the data splits we applied.

	Controlled dataset		Remote-uncontrolled dataset
	Train and validation	Test	Test
DM signals	12,992	12,115	1,172
SWD signals	16,857	13,560	21,572

A second set of wingbeat signals was compiled from data acquired in a different lab environment 6 months prior to the controlled dataset (Table 1). Data collection for this dataset lasted from late July until middle of October 2020, starting with the SWD class. The collection process of the DM class was initiated in August, but it was interrupted due to being provided with a non-flying variant of DMs. The process restarted late in September with a stock of wild DMs, but it was hindered by the environmental and room conditions at that time; hence the low numbers of DM wingbeat signals collected. Temperature and humidity were not controlled and varied according to the room environmental conditions, which were on average 23 ± 1°C and 55 ± 9% RH. After applying the higher mentioned filtering procedure, a total of 22,744 wingbeat signals were retained in this dataset; 21,572 of those belong to the SWD class and 1,172 belong to the DM class.

### Selected Data Types and Data Cleaning

**Figure 3:**
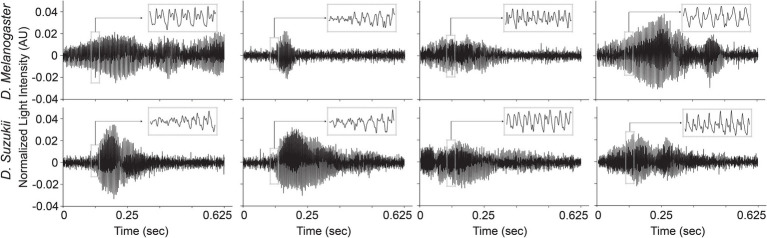
Illustrations of different wingbeat time signals of *Drosophila suzukii* and of *Drosophila melanogaster*.

**Figure 4:**
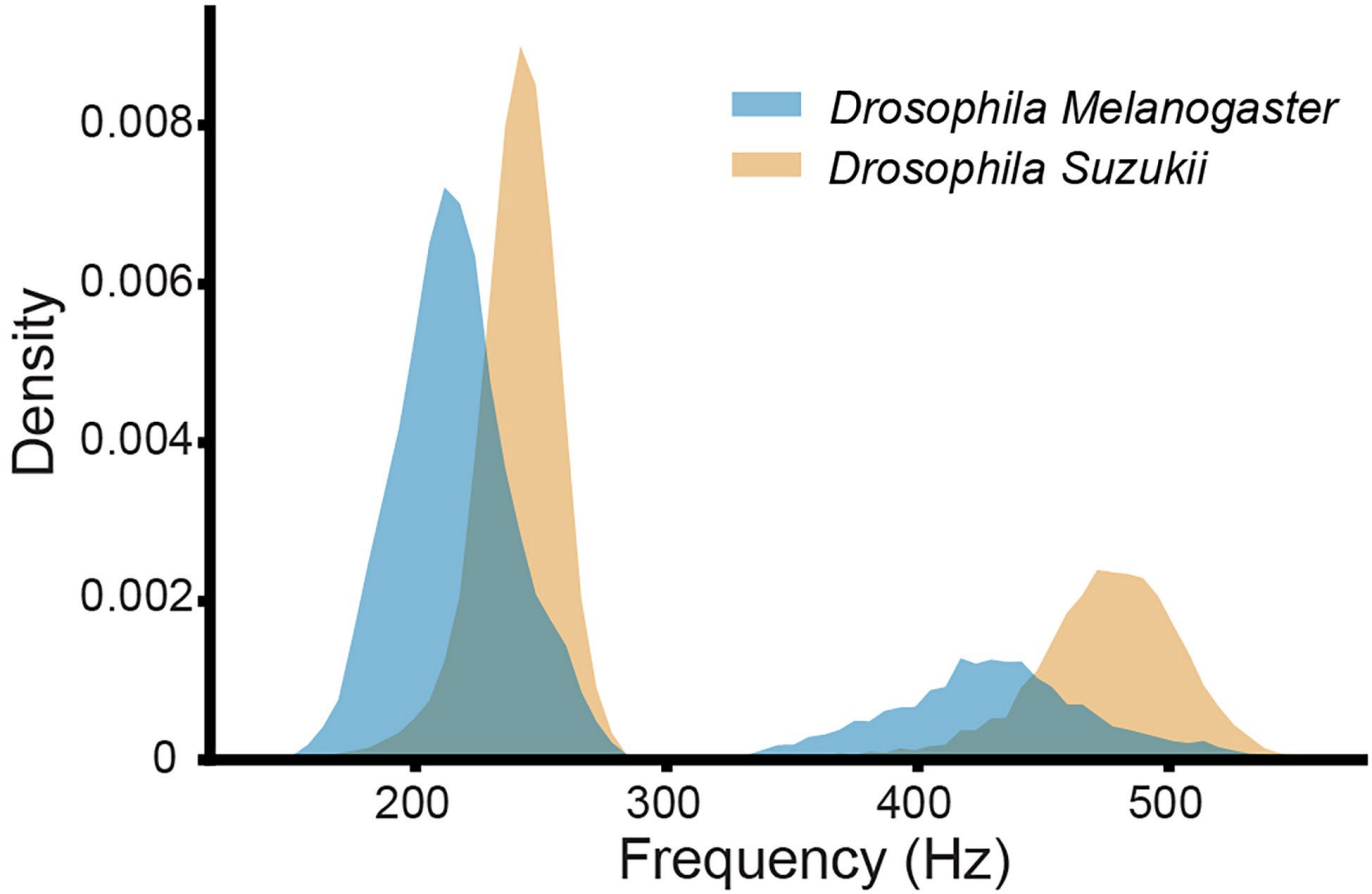
Histograms of the main wingbeat frequencies and the first harmonics of *D. melanogaster* and *D. suzukii* wingbeat signals from the controlled dataset.

The time profiles of the wingbeats collected by the optoelectronic sensor device were digitized using a sampling frequency of 8 kHz. According to the Nyquist-Shannon sampling theorem (Shannon, 1949), this value should be sufficient to cover the main wingbeat frequencies of most insects, which were estimated to be < 1 kHz (Byrne et al., 1988), and their respective overtones in fine detail. The recorded signals consist of 5,000 light intensity measurements across 0.625 s. The intra-class variability for the two insects’ wingbeat signals is high due to the various flight patterns that insects perform while flying through the sensor, while the inter-class difference seems small in both time (see Figure 3) and frequency domains (see Figure 4).

**Figure 5:**
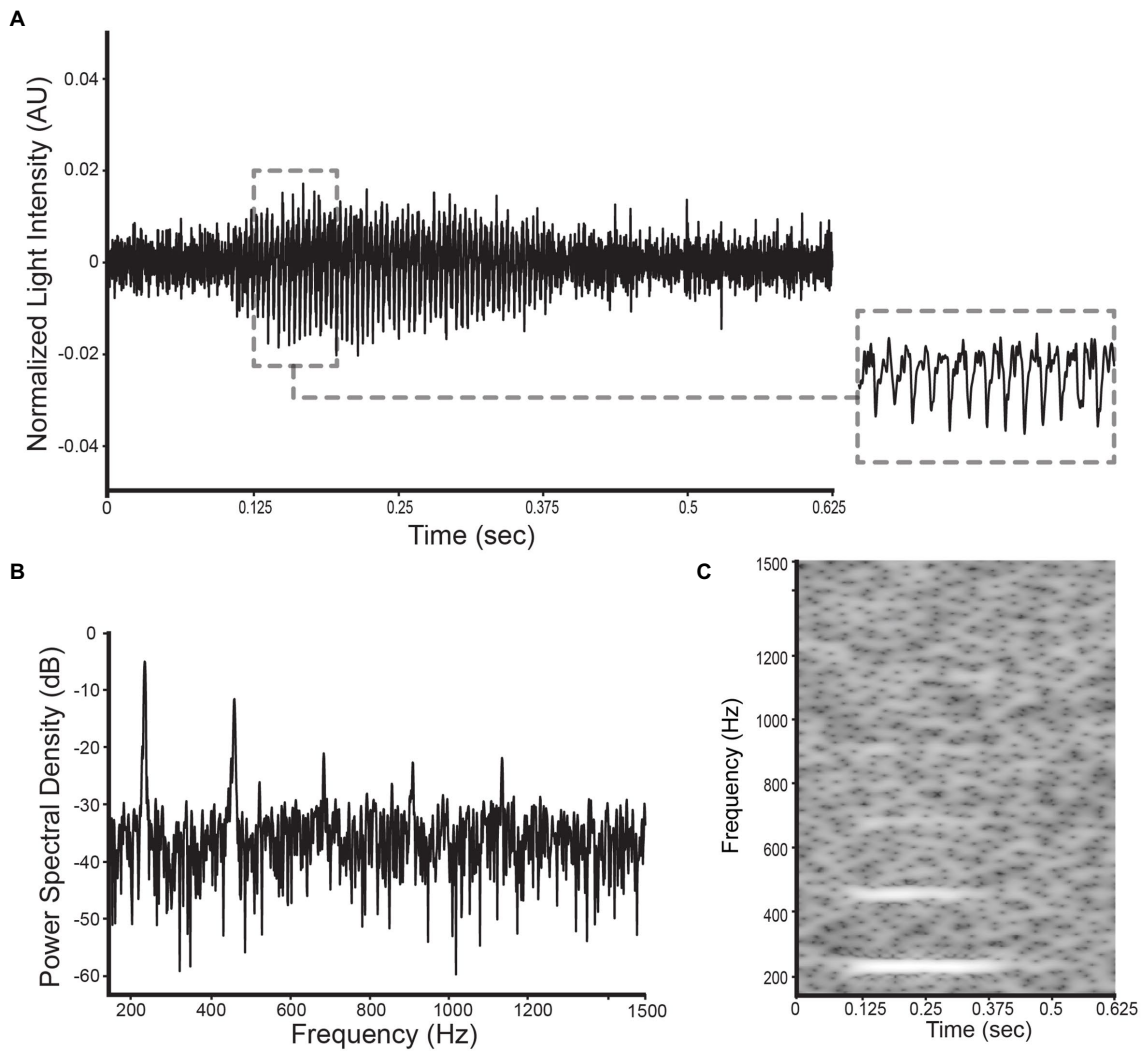
Illustration of the selected datatypes used in this study for a *D. suzukii* signal: **(A)** the wingbeat signal, **(B)** its power spectral density, and **(C)** its spectrogram.

The three data types that were analyzed and classified in this research are: (1) wingbeat time signals, (2) their frequency content, and (3) time-frequency content (see Figure 5). The frequency content of the wingbeat time signals was calculated using the Welch power spectral density (PSD) method with a “Hanning” window (FFT size of 8,192 samples, segment length of 5,000 samples and 2,500 samples overlap). The spectrogram of the wingbeat time signals is calculated as the frequency-over-time representation (FFT size of 8,192 samples, hopping length of 5, window length of 600).

A strict data cleaning procedure was employed to remove weak signals or false triggers captured by the sensor. A preprocessing bandpass filter was first applied to all signals (“low-cutoff”: 140 Hz, “high-cutoff”: 1500 Hz). Then, two metrics were employed to evaluate the validity of wingbeat signals: (a) a “PSD-score” defined as the sum of a wingbeat signal’s L2-normalized PSD values and (b) the number of peaks detected in its PSD, measured in V^2^/Hz. The peaks were detected using Scipy-library’s “find_peaks” function (McKinney, 2010) with the following settings:

- “prominence” = 0.001,
- “height” = 0.04,
- “width” = 1,
- “distance” = 5.

**Figure 6:**
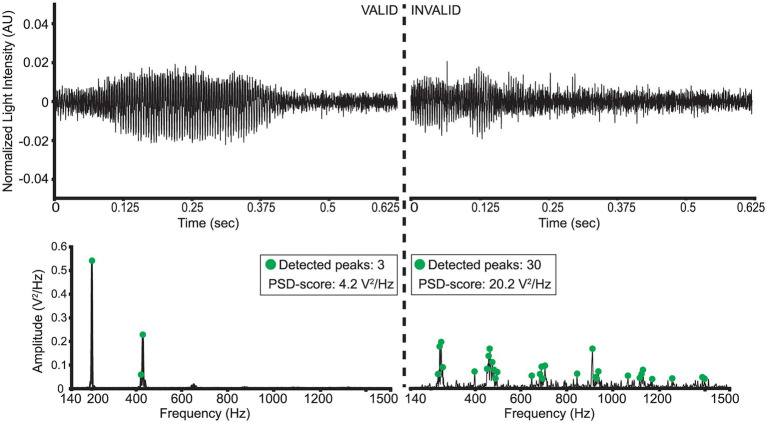
Illustration of a “valid” and “invalid” *D. melanogaster* wingbeat signal and their respective PSD’s.

A wingbeat signal was considered valid if its PSD-score was between 3.5 V^2^/Hz and 12 V^2^/Hz, and it had more than 1 but fewer than 15 peaks in its PSD. These threshold choices for the two metrics were found to substantially reduce the number of weak or noisy signals without discarding too much data. In theory, a clean wingbeat signal PSD is expected to contain five peaks in total—one peak at the main wingbeat frequency (max<300 Hz; see Figure 4) and a single peak for each of the occurring harmonics. In practice, however, more peaks might occur in a high-resolution PSD (see Figure 6). Therefore, a ceiling of maximum 15 peaks is considered to be a safe threshold to keep signals with three times more peaks in their PSD than the theoretically “cleanest” signal and remove noisier signals. Lowering this threshold did not have a significant impact on the resulting signals, so further optimization is possible, but its increase is not recommended. Examples of a valid *D. melanogaster* wingbeat signal and one that was rejected by the above procedure are shown in Figure 6. The bandpass-filtered wingbeat signals were then fed to the classification models as waveforms of 5,000 dimensions or as PSD and spectrogram transformations. Both the PSD and spectrogram data were converted to decibel (dB) scale and only the values within the preprocessing filter’s range (i.e., 140 to 1,500 Hz; 1,360 dimensions) were retained. The spectrogram images were downscaled to 295 × 400 pixel dimensions, maintaining the same aspect ratio of the original spectrograms, while allowing computational efficiency during training.

### Data Splitting and Performance Evaluation

The aim of this research was to design an experiment where it would be possible to validate our trained models in a strict way and uncover their “true” performance in field conditions. To this end, we used data from two different datasets. The “Controlled” dataset, where data was collected under controlled environmental conditions and a “Remote-Uncontrolled” dataset where environmental variables were not controlled, and the data acquisition was 6 months earlier than for the controlled dataset (Table 1).

It should be noted that in our experimental setting a single insect can produce multiple similar signals within a population, because it can fly through the sensor multiple times while in the enclosure. When a random validation strategy would be applied, these highly similar datapoints could end up in different data splits and lead to over-optimistic estimates for the model performance (Kalfas et al., 2021). Using separate populations for training and testing, we aimed to tackle this problem and uncover the models’ “true” classification performance which would emerge in field conditions. Hence, for the controlled dataset we created two separate insect populations for each of the two fruitfly species we are classifying (Figure 2). For each insect species, the population with the higher number of samples was chosen for training our models (“A” groups; Figure 2) and the other is used for testing (“B” groups; Figure 2). The training set was further split into training and validation sets which consist of 80 and 20% of its randomly sampled data, respectively. This validation set was used for hyper-parameter tuning of the models during training and model checkpoint selection. The remote uncontrolled dataset, which contains different insect populations, was used as an additional, truly external test set.

To evaluate the classification performance, we calculated the balanced accuracy and *F*1-score metrics on the test sets. The balanced classification accuracy in this binary setting is defined as the average of the proportion of correct predictions of each class individually, or the average of recall obtained on each class (best value equals to 1 and worst value is 0). The recall is defined as:


$$\mathrm{recall}=\frac{\mathrm{TP}}{\mathrm{TP}+\mathrm{FN}},$$

where TP is the number of true positives and FN the number of false negatives. To calculate the *F*1-score, we first define precision as:


$$\mathrm{precision}=\frac{\mathrm{TP}}{\mathrm{TP}+\mathrm{FP}},$$

where TP is the number of true positives and FP the number of false positives. Finally, the *F*1-score is defined as:


$$F1-\mathrm{score}=2*\frac{\mathrm{precision}*\mathrm{recall}}{\mathrm{precision}+\mathrm{recall}}$$

which constitutes the harmonic mean between precision and recall. Time required to train or perform inference is measured and compared across models. For the latter, we take the average of five runs given a single batch of size 1. For the model with the highest classification performance, we report its confusion matrix for the test sets derived from the Controlled and Remote-Uncontrolled datasets.

### Model Architectures and Training

Custom and state-of-the-art models from literature were chosen to fit 3 different types of wingbeat data, i.e., wingbeat time signals, their frequency (PSD) and time-frequency representations (spectrograms). For the wingbeat time and frequency signals, two models were trained: a custom 8-layer CNN—which we named “DrosophilaNet,” and a variation of the state-of-the-art model for time-series and 1-dimensional data classification known as “InceptionTime”(Fawaz et al., 2019)—which we named “InceptionFly.” DrosophilaNet consists of 8 blocks of Convolutional (“type”: 1D-Convolution, “activation”: ReLU), Batch-Normalization and Max-Pooling layers (“window”: 2) that progressively create lower dimensional representations of the original data and feed their output to an Average Pooling, a Dropout layer (“drop rate”: 0.2) and a Linear classification layer (“activation”: Sigmoid) with 1 output unit. The number of filters in the convolutional layers increased in powers of 2, starting from 16 in the 1st block, to 2,048 in the 8th block, while the kernel size was fixed to a value of 3.

InceptionTime consists of residual blocks which in turn consist of multiple “inception modules” each. The residual blocks’ input is transferred *via* skip connections to be added as input to the next block. Inception modules in each block reduce the input’s dimensionality using a bottleneck layer and then extract hierarchical features of multiple resolutions by applying convolution filters of various lengths in parallel. These features are pooled, convolved, batch-normalized and fed to a ReLU activation function. For our InceptionFly, we used two residual blocks composed of three inception modules each. All inception modules had a fixed number of 32 convolutional filters using kernel sizes of: 6, 12, and 24. The two residual blocks were followed by an Average Pooling layer and a Linear classification layer (“activation”: Sigmoid).

**Figure 7:**
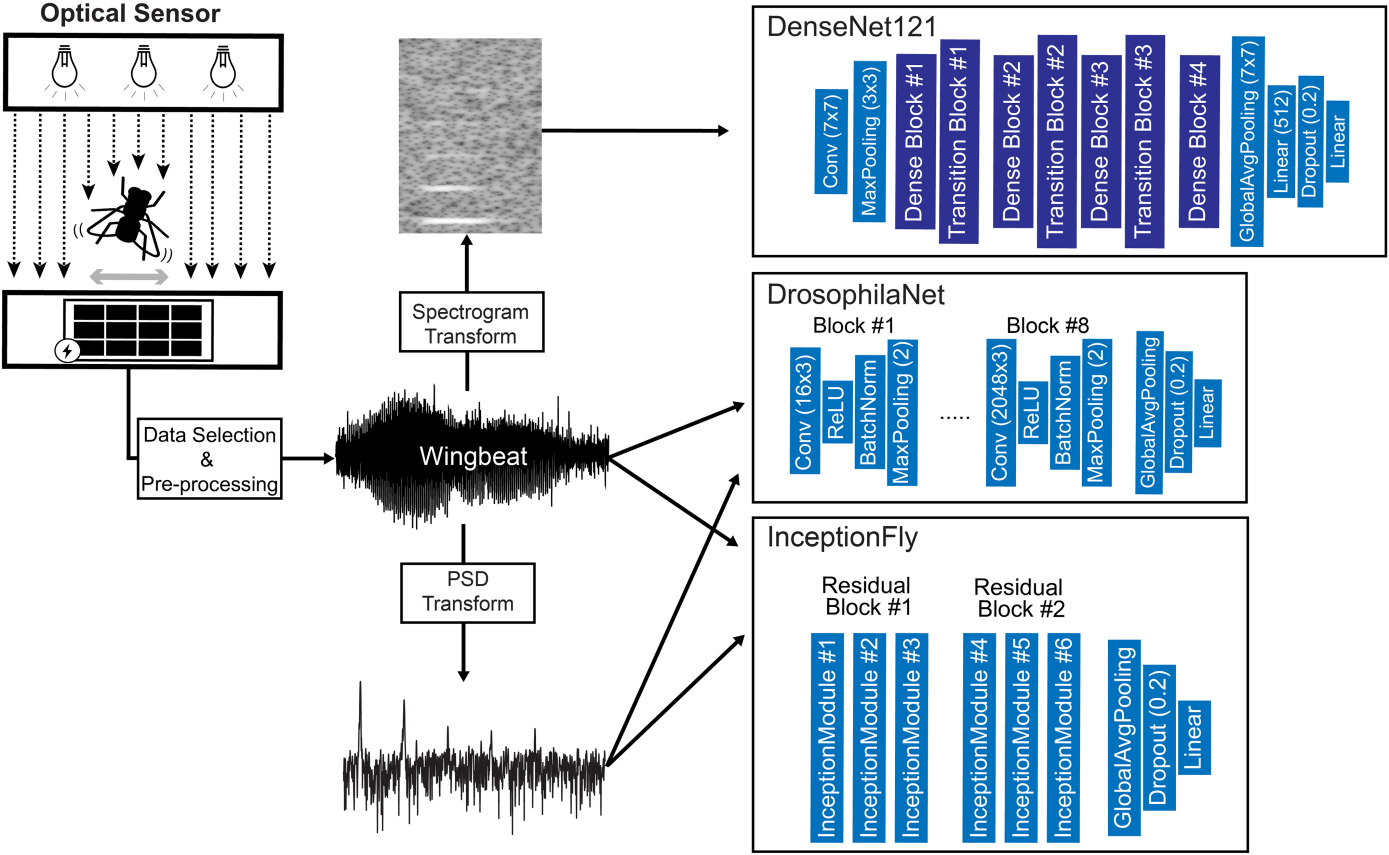
Diagram of the data processing and modeling procedures including an illustration of the optical wingbeat sensor and the model architectures used in this study. For more information on the “Dense Block” and “Transition Block” layers, see Huang et al. (2017).

The spectrogram images were modeled with DenseNet121 (Huang et al., 2017), which is a popular CNN model for image classification tasks that was already tested and known to perform well in a similar task of classifying mosquito spectrogram images (Kalfas et al., 2021), while ranking first among other popular CNN models in a different study (Fanioudakis and Potamitis, 2018). We removed the top layer of DenseNet121 to replace it with a Linear fully-connected layer with 512 units (“activation”: ReLU), a Dropout layer (“drop rate”: 0.2) and a Linear classification layer (“activation”: Sigmoid) with 1 output unit. Its input layer dimensions were modified to match our spectrogram data dimensions (295 × 400) and the rest of the model’s architecture remained intact. A summary of our data processing pipeline and an illustration of the model architectures used, are presented in Figure 7.

The training procedure for all neural network models was designed with the following settings:

- Training epochs: 100.
- Batch size: 32.
- Loss: categorical cross-entropy.
- Optimizer: Adam.

To help the neural networks to converge faster and reach high classification rates we used Cyclical Learning Rates (CLR; Smith, 2017) with the following settings for the CLR scheduler:

- Base learning rate: 0.0001.
- Max learning rate: 0.01.
- Cycle momentum: False.
- Mode: triangular.

The training procedure was allowed to run for 100 epochs while saving a model checkpoint (with the model’s parameters) in each epoch. In the end, we selected the model checkpoint that showed the maximum validation accuracy. This accuracy is different from the balanced accuracy score we report on the model performance and is defined as the set of labels predicted by the model for each training datapoint, that exactly match the corresponding ground truth labels.

All models output a single probability score, ranging from 0 to 1, based on the Sigmoid activation of their last Linear classification layer. Probability scores below 0.5 are mapped to DM predictions, while scores greater or equal to 0.5 indicate a SWD prediction. Thus, in this binary classification setting the DM is considered the “negative class” and SWD the “positive class.” We fine-tuned the selected models’ decision thresholds by choosing the threshold that maximized the respective model’s balanced accuracy score on the validation data (Fernández et al., 2018).

While training our models we experimented with custom data-augmentation techniques to increase model robustness and guide the neural networks in learning the important distinguishing features of the input data. Since all analyses begin with the wingbeat time signals—which are either modeled directly or transformed into frequency (PSD) or time-frequency (spectrogram) representations—we designed data transformations that might be applied on them as an “online” pre-processing step. First, a “Random-Roll” operation was applied that shifts the raw signal forwards or backwards in time by a number (of samples) randomly chosen from a range between 500 (0.0625 s) and 4,500 (0.5625 s). The part of the time signal that goes out of the original length because of shifting forwards (or backwards) is attached at the beginning (or the end) of the time signal. This augmentation technique helps in producing signals for various insect flights. Second, a “Random-Flip” operation was applied which mirrors the signal in the time dimension and third a “Random-Noise” operation was applied which adds Gaussian noise in a randomly selected part of the signal, which acts like signal “time masking” (Bouteillon, 2019). Each of the above operations had a 50% chance to be applied to any given input signal during training. As these 50% changes were applied independently, combinations of these operations were also possible.

All experimental scripts to train, evaluate and visualize our results were written in Python3, using the Pytorch library (version 1.8.1), Scikit-learn (version 0.24.1), and other scientific computing libraries (McKinney, 2010; Oliphant, 2010; Pedregosa et al., 2011; Mcfee et al., 2015). The code was executed on a single GPU (Nvidia RTX 5000; 16 GB RAM) laptop computer.

## Results and Discussion

### Wingbeat Signals

As illustrated in Figure 4, the main wingbeat frequencies and the first harmonics of SWD and DM overlap. This makes it difficult to use these features for efficiently classifying between SWD and DM (Chen et al., 2014; Genoud et al., 2018). There is also no clear distinction between the two sexes of either insect species in terms of their wingbeat frequencies. This is not unexpected since visually, the sexes of both *Drosophila* species are very similar. Having a highly similar wing and body shape is expected to result in highly similar wingbeat recordings, which is confirmed by the wingbeat time signals for SWD and DM in Figure 3. Sex and age have been reported to influence the wingbeat recordings (Chen et al., 2014; Genoud et al., 2018). However, such information was not included in this study as for each *Drosophila* species both male and female flies of varying age were placed in the cages with the optical sensor, as would be the case in the field.

Our data cleaning procedure retained 55,524 valid wingbeat signals in the controlled dataset. Out of those, 29,849 were used for training and validation (SWD: *n* = 16,857; DM: *n* = 12,992), and the remaining 25,675 signals formed the test set (SWD: *n* = 13,560; DM: *n* = 12,115). For the remote uncontrolled dataset, the data cleaning procedure retained 22,744 valid wingbeat signals. Out of those, 21,572 belonged to SWD and 1,172 to the DM class. The low number of DM wingbeat signals in the Remote-Uncontrolled dataset can be attributed to unfavorable external conditions during this experiment. The experimental setup was in the same room as other machinery that raised the temperature and dried up the air during the morning hours of the same time period. This motivated us to use climate chambers for the collection of the Controlled dataset. Notably, the data acquired from the DM cages contained a considerably higher number of invalid signals compared to the SWD data. This may partly be attributed to the higher activity levels of DMs that lead to falsely triggering the sensor more often, e.g., by crawling on the sensor head. SWD insect population sizes seemed more stable throughout the length of the experiment, in contrast to DM populations which seemed to fluctuate.

### Classifier Performance

**Figure 8:**
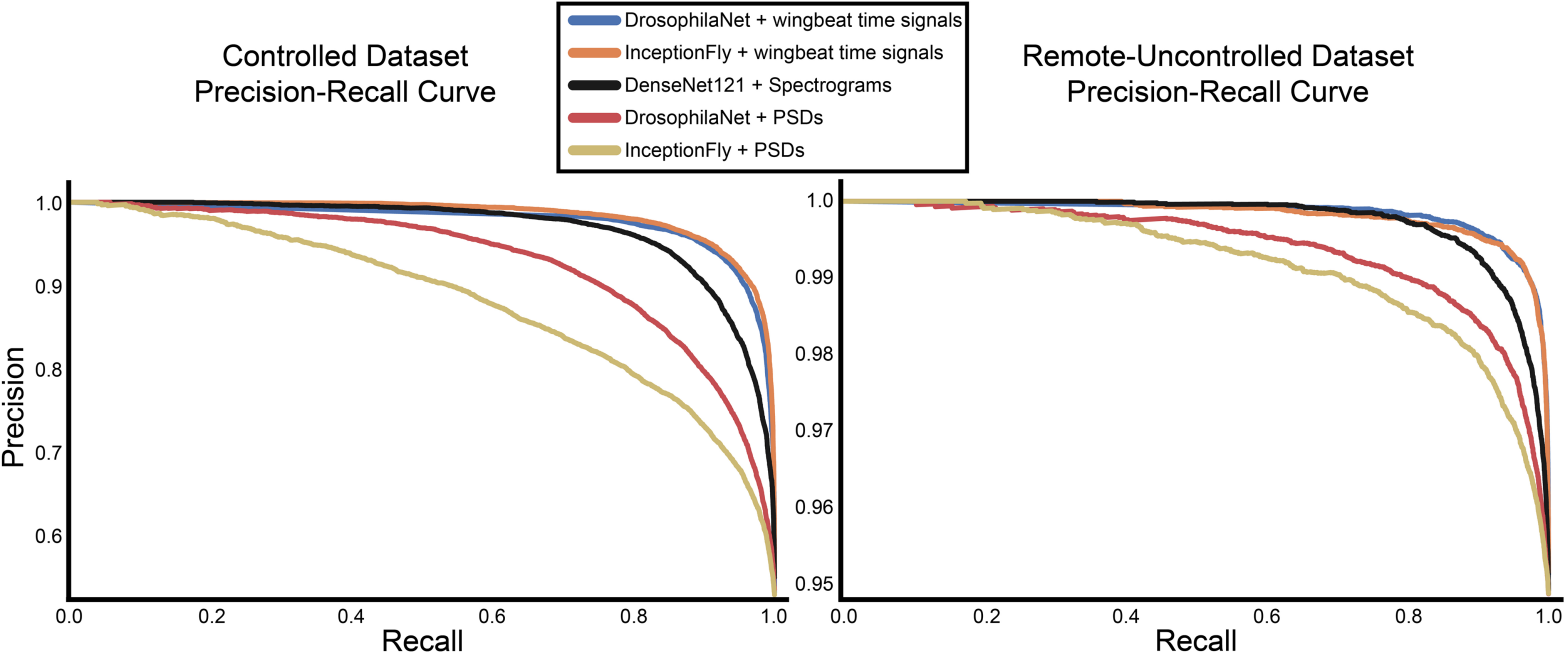
Precision-recall curves for all models for the controlled and remote-uncontrolled datasets.

**Figure 9:**
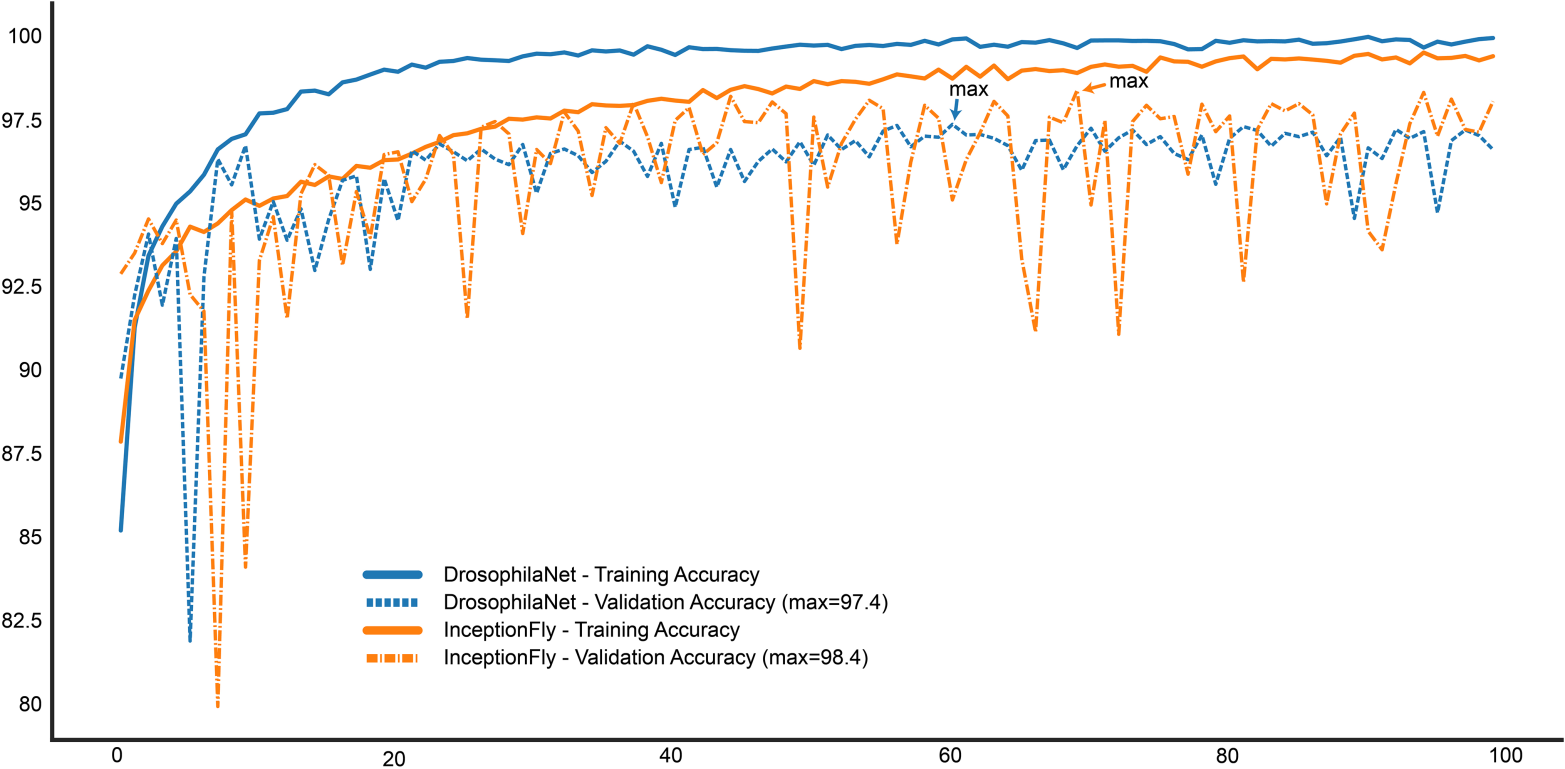
Training and Validation accuracy curves for the top two performing models: InceptionFly and DrosophilaTime.

The performance of all classifiers is summarized in Table 2. Their precision-recall curves for both datasets are shown in Figure 8. The best performing model was InceptionFly with wingbeat time signals. Trained with the Controlled dataset, it classifies wingbeat signals from the Controlled test set with a balanced accuracy score of 92.1% and *F*1-score of 0.93. DrosophilaNet performed similarly with a balanced accuracy of 91% and *F*1-score of 0.92. Using either InceptionFly or DrosophilaNet with PSD input data provided inferior classification results with balanced accuracies of 78.7 and 81.8%, and *F*1-scores of 0.67 and 0.84, respectively. Densenet121 trained with spectrograms provided a balanced accuracy of 87% and *F*1-score of 0.80 in the Controlled test set. This is in line with our previous work, where “InceptionTime” outperformed all other models on either wingbeat time signals, frequency signals or time-frequency signals (Fawaz et al., 2019; Kalfas et al., 2021). However, in this study, we found that DrosophilaNet had similar performance while being faster to train and perform inference with, compared to InceptionFly. In Table 2 and Figure 8, we note that DrosophilaTime is more capable to model PSD data in both datasets, while it trains and performs inference on it faster, too. In Figure 9, the training and validation accuracy curves are plotted for the top two models in classification performance – InceptionFly and DrosophilaNet trained with wingbeat time signals. Despite InceptionFly reaching a higher validation accuracy, DrosophilaNet converges faster in the training set, while showing signs of high validation accuracies from the 10^th^ epoch onwards. This makes it a good candidate for being deployed in the field where fast training and inference are critical. However, it could be interesting to investigate simpler variants of InceptionFly—fewer filters or smaller kernel sizes – that could improve its training and inference time performance.

**Table 2:** Model performance for selected data types on the two test datasets (controlled and remote-uncontrolled).

Input	Model	Decision threshold	Total training time	Inference time	Controlled dataset		Remote-uncontrolled dataset	
					Balanced accuracy	*F*1-score	Balanced accuracy	*F*1-score
PSD (1360 × 1)	DrosophilaNet	0.694	**31 min**	**4 ms**	81.8%	0.84	83%	0.90
	InceptionFly	0.674	1.25 h	5.5 ms	78.7%	0.67	79.5%	0.86
Wingbeat signal (5000×1)	DrosophilaNet	0.744	53 min	4.8 ms	91%	0.92	91%	**0.97**
	InceptionFly	0.737	3.3 h	7.8 ms	**92.1%**	**0.93**	**91.6%**	0.96
Spectrogram (295×400)	DenseNet121	0.646	36.6 h	29.8 ms	87%	0.80	88.1%	0.95

Classification performance is measured using the balanced accuracy and *F1*-score. The models’ fine-tuned decision thresholds are reported along with the total training time (measured in minutes or hours) and the inference time which was estimated for a batch size of 1, by taking the mean inference time of five runs for each model (measured in milliseconds). The best score for each performance metric is shown in bold.

**Figure 10:**
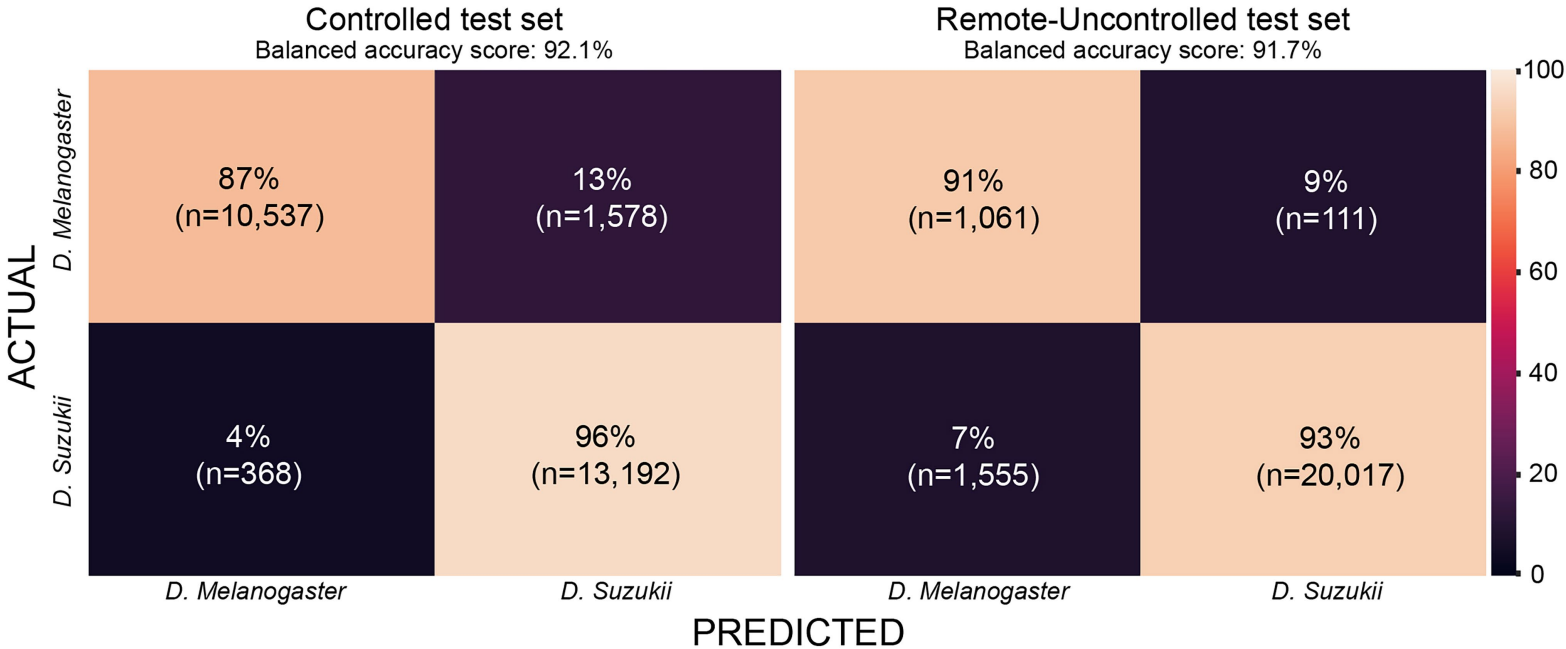
Confusion matrix for InceptionFly trained with wingbeat time signals for our two datasets.

The Remote-Uncontrolled dataset was used as an additional test set to evaluate our models’ robustness. In Figure 10, the best model’s confusion matrix and classification performance using wingbeat time signals on this dataset are illustrated. Data belonging to this dataset were collected months in advance, in different environmental conditions – which were expected to be closer to in-field conditions, and from different insect populations compared to those included in the training set. Still, InceptionFly trained on the Controlled dataset was able to classify wingbeat time signals in this Remote-Uncontrolled dataset with a balanced accuracy score of 91.6% and *F1*-score of 0.96. DrosophilaNet was again a close second with a balanced accuracy of 91% and a slightly higher *F*1-score of 0.97.

The two classification performance metrics used in this study—balanced accuracy and *F1*-score—are both reliable metrics for binary classification problems, but they are not equally sensitive to how the model performs on both classes. The *F*1-score is more sensitive to a model’s performance in the positive class (SWD), while balanced accuracy equally considers both classes (SWD and DM) when evaluating model performance. This means that a higher *F*1-score is expected when a model accurately classifies many SWD signals regardless of making more mistakes in the DM predictions. On the other hand, the balanced accuracy metric assigns equal weight to SWD and DM mistakes. This explains the high *F*1-scores for the class-imbalanced Remote-Uncontrolled dataset. From a pest monitoring perspective, one could argue that it is more important to classify SWD correctly, but a robust model should also be sensitive to the DM classification performance for both the Controlled and Remote-Uncontrolled dataset. Therefore, we report both metrics.

Models trained on wingbeat time signals outperformed models using either PSD or spectrograms as input on both datasets. This suggests that important information for classifying the wingbeats of these two highly similar insect species is present in the time dimension. It is hypothesized that micro-movements of the insects’ wings are captured by the artificial neurons of InceptionFly or DrosophilaNet, which helps them classify wingbeats more accurately. This information is likely averaged out in the PSD and spectrograms. Higher resolution spectrograms could lead to better classification results, but that would create higher computational costs with even longer training and inference times. Besides, DenseNet121 was already the slowest among all models requiring 36.6 h to train and 29.8 ms to perform inference on a single datapoint, which is, respectively, 12 and 4 times longer than for the best performing model InceptionFly (see Table 2).

### Towards Deployment in the Field

To obtain more insight in the cases were the algorithms resulted in misclassifications, we analyzed the temperature, relative humidity and timestamp of all misclassified wingbeat recordings. However, no clear correlations were found between these parameters and the models’ classification performance. To obtain a better understanding of where the model fails and in what aspects the wingbeat patterns of the two species differ, it is recommended to investigate the role of the sampling frequency on classification performance of deep CNNs and to focus on the models’ explainability.

The results reported here were obtained without applying any of the aforementioned data augmentation techniques since no significant performance change was noted when using these. Similar classification results were reached with all different data types used in this research when employing one or a combination of all considered data augmentation techniques. Data augmentation is expected to have a stronger effect when used with much smaller amounts of data since it would help to capture all different variations of the input data that would remain unseen given less data. An interesting follow-up study could help to identify the classification performance of wingbeat models and the effect of data augmentation starting from few data and increasingly adding more. The non-deterministic nature of neural networks would need to be taken into account when performing such experiments, since slight performance changes are expected after every training procedure.

The confusion matrix for InceptionFly trained with wingbeat time signals indicates a strong classification ability for this model (Figure 10). InceptionFly seemed to perform better for the SWD class compared to the DM class, since for the Controlled test set, only 4% of all SWD samples were misclassified as DM compared to 13% for the DM samples. For the Remote-Uncontrolled test set the misclassification rates were more balanced with 7 and 9%, respectively. The in-field performance of InceptionFly is expected to be close to its performance on the Remote-Uncontrolled dataset, but some challenges are expected still due to variation in the wingbeat frequencies in response to variable environmental conditions (Unwin and Corbet, 1984). Therefore, special attention needs to be given to performance monitoring and error analysis when the model is deployed in the field, especially for signals collected in extreme environmental conditions that were not covered in our two datasets.

## Conclusion

Fruit production is increasingly challenged by the *D. suzukii* fruitfly which lays its eggs in healthy ripening fruits rather than damaged or overripe ones. Fruit growers demand automatic monitoring tools to efficiently protect fruit crops against this pest. To this end, we combined an optical wingbeat sensor with convolutional neural networks and evaluate the possibility to discriminate the wingbeat signals acquired for *Drosophila suzukii* and *D. melanogaster* fruitflies. To our knowledge, no other studies have previously built classification models for these two common pests. All models used in this work were validated in a strict way to uncover the “true” classification performance that can be expected in field conditions. A first validation involved classification of wingbeat signals collected in different enclosures under the same environmental conditions. Our best performing model, InceptionFly trained with wingbeat time signals was able to discriminate these wingbeat signals with an accuracy of 92.1%. Next, the model was also validated on wingbeat signals that had been collected independently under more variable environmental conditions. This validation was also successful with an accuracy of 91.7%. This shows that this model is sufficiently robust to be embedded in an automatic insect monitoring system that will operate in field conditions to provide accurate estimates of *D. suzukii* and *D. melanogaster* pest presence.

## Data Availability Statement

The raw data supporting the conclusions of this article will be made available by the authors on request, without undue reservation.

## Author Contributions

IK: conceptualization, methodology, software, validation, formal analysis, investigation, data curation, writing—original draft, writing—review and editing, and visualization. BK: conceptualization, methodology, resources, writing—review and editing, project administration, funding acquisition, and supervision. TB: resources, writing—review and editing, project administration, and funding acquisition. WS: conceptualization, methodology, resources, writing—review and editing, project administration, and supervision. All authors contributed to the article and approved the submitted version.

## Funding

This research conducted with the financial support of VLAIO (Flanders Innovation & Entrepreneurship) (project HBC.2016.0795) and the Horizon 2020 Research and Innovation Programme, Grant Agreement no. 862563.

## Conflict of Interest

The authors declare that the research was conducted in the absence of any commercial or financial relationships that could be construed as a potential conflict of interest.

## Publisher’s Note

All claims expressed in this article are solely those of the authors and do not necessarily represent those of their affiliated organizations, or those of the publisher, the editors and the reviewers. Any product that may be evaluated in this article, or claim that may be made by its manufacturer, is not guaranteed or endorsed by the publisher.
